# Correlation of Psoas Muscle Index with Fragility Vertebral Fracture: A Retrospective Cross-Sectional Study of Middle-Aged and Elderly Women

**DOI:** 10.1155/2022/4149468

**Published:** 2022-11-02

**Authors:** Yihui Zhang, Yilihamu Dilixiati, Wei Jiang, Xiufeng Cao, Yuanyuan Chen, Hui Guo

**Affiliations:** ^1^Department of Radiology, Sixth Affiliated Hospital of Xinjiang Medical University, Urumqi 830002, China; ^2^Department of Orthopedic, Sixth Affiliated Hospital of Xinjiang Medical University, Urumqi 830002, China; ^3^Medical Imaging Center, First Affiliated Hospital of Xinjiang Medical University, Urumqi 830054, China

## Abstract

**Objective:**

To investigate the correlation of psoas muscle index (PMI) with fragility vertebral fracture.

**Methods:**

A total of 184 middle-aged and elderly women were included in the study. We measured the bilateral psoas muscle area on the picture archiving and communication system (PACS) from computed tomography images and calculated PMI. We observed lateral radiographs of the thoracolumbar spine and assessed vertebral fractures using the Genant semiquantitative method. The *T*-score, bone mineral density (BMD) of the lumbar (L)1-4, femoral neck, and trochanter were measured by dual-energyX-ray absorptiometry (DXA). The data was collected and then statistically analyzed.

**Results:**

The PMI of the nonosteoporosis group was higher than that of the osteoporosis group (*P* value = 0.006). Height in the nonosteoporosis group was higher than that in the osteoporosis group (*P* value = 0.013). Weight, body mass index (BMI), left psoas muscle area, BMD of the L1-4, femoral neck, femoral trochanter, and *T*-score in the nonosteoporosis group were higher than those in the osteoporosis group (*P* value <0.001). The right psoas muscle area in the nonosteoporosis group was higher than that in the osteoporosis group (*P* value = 0.008). The incidence of combined thoracolumbar fracture was significantly higher in the osteoporosis group than that in the nonosteoporosis group (*P* value <0.001). For nonosteoporosis subjects, the PMI of the vertebral fracture group was lower than that of the nonvertebral fracture group (*P* value = 0.034).

**Conclusions:**

A decrease in height, weight, BMI, bilateral psoas muscle area, and PMI is associated with osteoporosis. Combined thoracolumbar fractures are more common in osteoporosis. Sarcopenia may be an independent risk factor for nonosteoporotic vertebral fractures.

## 1. Introduction

Musculoskeletal disorders have become significant public health problems as the population ages. Irwin Rosenberg first used sarcopenia to define sarcopenia in 1989, and since then, muscle function has become essential in describing sarcopenia [1]. Sarcopenia is a progressive decrease in body muscle mass and/or a decrease in muscle strength or muscle physiological function associated with age. The relationship between sarcopenia and osteoporosis has increasingly become a hotspot, and significant progress has been made in clinical and basic research [2–4]. Vertebral fracture is considered a sign of osteoporosis and is the most common fragility fracture. It is of great significance to study the relationship between paraspinal muscles and thoracolumbar osteoporotic fractures and to further early intervention treatment for diagnosing and treating thoracolumbar vertebral compression fractures.

Psoas muscle index (PMI) is used to evaluate skeletal muscle mass through computed tomography (CT). PMI is positively correlated with total skeletal muscle volume and can be used to diagnose sarcopenia [5, 6]. A recent study showed that PMI measured by CT in patients with spinal degeneration was positively correlated with bone mineral density (BMD). It was considered a useful tool for assessing osteoporosis and fracture risk [7]. Bilinc Dogruoz Karatekin suggested that PMI may be associated with hip fractures in patients with osteoporosis. Selective psoas strengthening exercises may be beneficial for hip fracture prevention and posthip fracture rehabilitation program [8]. Is sarcopenia related to fragility vertebral fracture? This retrospective cross-sectional study explores the association between sarcopenia and fragility vertebral fracture using PMI.

## 2. Materials and Methods

### 2.1. Subject

214 women who visited the Sixth Affiliated Hospital of Xinjiang Medical University from January 2021 to December 2021 were enrolled. Inclusion criteria: women over 50 years of age with clinical syndromes of back pain and limited spinal mobility, with aninterval among CT, spinal radiographs, and BMD examination within 3 months. Based on imaging and medical history, we excluded 12 cases with vertebral fractures caused by a high-energy trauma or degenerative disease based on the qualitative criteria used in clinical practice for differential diagnosis of vertebral fractures; 2 cases who had taken calcitriol or alendronate sodium tablets for 3 months; 1 case who had hypothyroidism; 3 cases of severe lumbar degeneration; 4 cases of pathological vertebral fracture; 1 case of septic spondylitis; 5 cases of spinal tuberculosis; 2 cases of brucella spondylitis. Finally, 184 women aged 55–90 (70.5 ± 7.4) years were included in the study.

We collected clinical data from the subjects including age (y), menopausal age (y), height (m), weight (kg), and history of fractures. Weight divided by the square of height is the body mass index (BMI) (kg/m^2^) calculation formula.

### 2.2. Measurement of Bone Mineral Density

BMD of the lumbar spine and one side of the hip joint was measured by dual-energy*X*-ray absorptiometry (DXA) (GE, Lunar Prodigy). Measurement process: the subjects lay supine in the middle of the examination table with their hands flat on both sides of their bodies. The posterior-anterior program scanned the spine and hip joints. After scanning, the software automatically generates data and measurement reports. The collected data included BMD of lumber (L)1-4, femoral neck, femoral trochanter, and *T*-scores. The L1-4 vertebrae, femoral neck, and femoral trochanter were used as regions of interest (ROI), and the lowest *T*-score of the 6 ROIs was used to diagnose osteoporosis. According to the DXA osteoporosis diagnostic criteria recommended by the academic organization of osteoporosis in China [9], participants were defined as osteoporosis (T-score ≤−2.5 standard deviations (SD)), osteopenia (−2.5 SD < *T*-score ≤ −1 SD), and normal bone mass (T-score > −1 SD).

### 2.3. Evaluation of Vertebral Fracture and Psoas Muscle Index

All subjects underwent lateral radiographs of the thoracic (T) 4-L5. One radiologist read radiographs and determined vertebral fractures according to the Genant semiquantitative method [10]. The criterion for vertebral fracture was a reduction of more than 20% in the anterior, middle, or posterior vertebral height. The site of the fracture was recorded.

**Figure 1:**
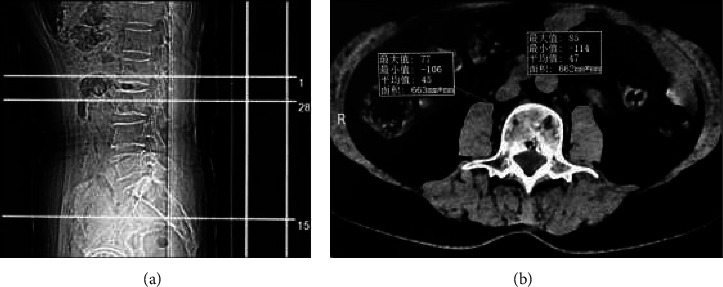
The two images are the CT scan localization image and the CT axial image of a 58-year-old woman with a height of 1.6 m, respectively. (a) The localization image showed that plane of the line 28 was the middle level of the L3 vertebral body. (b) One radiologist manually delineated the edge of the bilateral psoas muscle on the CT axial image of the L3 level. The bilateral psoas muscle area was calculated automatically by the software. The right psoas muscle area was 663 mm^2^, and the left psoas muscle area was 662 mm^2^. According to the calculation formula, the PMI was equal to 517.6 mm^2^/m^2^.

T12 to sacral (S)1 vertebral body of subjects were scanned by a 128-slicedual-source CT scanner (Siemens, Germany). CT scanning parameters are as follows: tube voltage 110 kV, tube current 100 mA, and layer thickness 1 mm. One radiologist who was blinded to BMDof the subjects selected CT axial images of the L3 vertebrae by picture archiving and communication system (PACS) (Heart Shadow International), and then manually delineated the border of the bilateral psoas muscle. The software automatically calculated the bilateral psoas muscle area, as shown in Figures 1(a) and 1(b). Total psoas muscle area divided by the square of height is the PMI (mm^2^/m^2^) calculation formula.

### 2.4. Statistical Analysis

We used the Statistical Product and Service Solutions 19.0 software package for statistical analysis. Measurement data were expressed as the mean ± standard deviation. Enumeration data were expressed as numbers (percentages). A Kolmogorov–Smirnov test was used to test whether the data conformed to a normal distribution. The homogeneity of variance for the measurement data was assessed using a Levene test. Measurement data of the two groups were compared using an independent sample *t*-test. A comparison of sample rates between the two groups was performed using a chi-square test or Fisher's exact test. *P* value < 0.05 was considered statistically significant.

## 3. Results

### 3.1. Baseline Data

There were 89 cases aged 56–86 (71 ± 6.8) years in the osteoporosis group. There were 95 cases aged 55–90 (70 ± 8) years in the nonosteoporosis group, including 62 cases with osteopenia and 33 cases with normal bone mass.

All subjects were postmenopausal women. Height in the nonosteoporosis group was higher than that in the osteoporosis group (*P* value = 0.013). Weight, BMI, left psoas muscle area, BMD of the L1-4 vertebral body, femoral neck, femoral trochanter, and *T*-score in the nonosteoporosis group were higher than those in the osteoporosis group (*P* value <0.001). The right psoas muscle area in the nonosteoporosis group was higher than that in the osteoporosis group (*P* value = 0.008). The PMI in the nonosteoporosis group was higher than in the osteoporosis group (*P* value = 0.006). There was no significant difference in age, age of menopause, or the incidence of vertebral and other fractures (humerus fracture, forearm fracture, femur fracture, and patella fracture) between the two groups (*P* value = 0.355, *P* value = 0.185, *P* value = 0.117, *P* value = 0.484, *P* value = 1, *P* value = 0.674, and *P* value = 0.233, respectively), as shown in Table 1.

### 3.2. Correlation between Psoas Muscle Index and Fragility Vertebral Fracture

**Figure 2:**
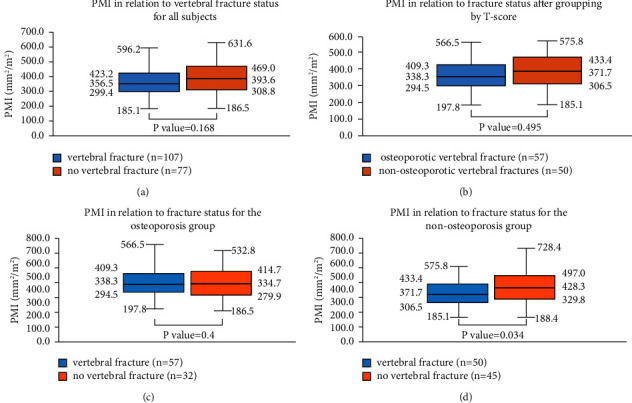
(a) The box plot showed the correlation between PMI and fracture status in all subjects. (b)∼(d) The three box plots showed the correlation between PMI and fracture status after grouping by *T*-score. The top-to-bottom data next to each box plot were the maximum, upper quartile, median, lower quartile, and minimum values of PMI. “*n*” indicates the number of cases in each group.

Of all participants, 107 cases had vertebral fractures including 57 osteoporotic and 50 nonosteoporotic. For the nonosteoporotic vertebral fracture group, 37 had osteopenia and 13 had normal bone mass. There was no significant difference between the PMI of the vertebral fracture group and that of the nonvertebral fracture group (*t* value = −1.384 and *PP* value = 0.168), as shown in Figure 2(a), and there was no significant difference between the PMI of the osteoporotic vertebral fracture group and that of the nonosteoporotic vertebral fracture group (*t* value =−0.684 and *P* value = 0.495), as shown in Figure 2(b).

For subjects with osteoporosis, there was no significant difference between the PMI of the vertebral fracture group and that of the nonvertebral fracture group (*t* value = 0.846 and *P* value = 0.4), as shown in Figure 2(c). For subjects with nonosteoporosis, the PMI of the vertebral fracture group was lower than that of the nonvertebral fracture group, and the difference was statistically significant (*t* value = −2.155 and *P* value = 0.034), as shown in Figure 2(d).

We divided the subjects into three categories according to the fracture site. The result showed that the incidence of combined thoracolumbar fracture was significantly higher in the osteoporosis group than that in the nonosteoporosis group (chi-square value = 12.331 and *P* value < 0.001), as shown in Table 2.

## 4. Discussion

Sarcopenia and osteoporosis are age-related declines in the quantity and quality of muscles and bones. Yeung et al. demonstrated that sarcopenia had positive correlation with fall and fractures in the elderly through a meta-analysis [11]. Zanchetta et al. found that sarcopenia was associated with increased fall risk, osteoporosis, and vertebral fractures in postmenopausal women [12]. The participants in this study were middle-aged and elderlywomen. Unlike in men, BMD in women is susceptible to estrogen levels. Compared with men of the same age, postmenopausal women are more likely to develop osteoporosis and have a higher risk of fragility fractures due to a sharp drop in estrogen levels [13, 14].

CT is the preferred method for quantitative assessment of sarcopenia. Skeletal muscle index (SMI) and PMI are the more commonly used indicators in research. SMI was calculated as the total skeletal muscle area at the level of the L3 vertebrae divided by the square of the height. The calculation method of PMI is similar to that of SMI. The calculation of PMI uses the psoas muscle area rather than the total skeletal muscle area. Our study used PMI as an assessment tool for sarcopenia, not only because of its ease of calculation but also to the anatomical function of the psoas muscle. As we all know, the psoas muscle adjacent to both sides of the lumbar spine is different from other trunk muscles, and it plays a crucial role in maintaining the upright posture of the human body. The psoas muscle connects the trunk and lower extremities to maintain standing and walking functions. Impairment of its function can seriously reduce the stability of body posture while affecting the human body's ability to flex the hip or maintain a standing posture. PMI is often used in research on Asian countries, especially Japan and South Korea [15–17]. A new PMI-based criterion for skeletal muscle mass has been established using data from healthy young Asian adults, which defines a cut-off value for sarcopenia in Asian populations [18]. In addition to evaluating sarcopenia, PMI can predict long-term mortality in young men with chronic or acute liver failure [19]. Several other studies have shown that PMI can be applied to Marfan syndrome and the prognosis of different tumor surgeries [20, 21].

Both sarcopenia and osteoporosis share the same pathophysiological basis and have a similar adverse effect on the health of older adults. Our results showed that the bilateral psoas area and PMI of postmenopausal women without osteoporosis were higher than those in the osteoporosis group. It indicates that reduction in skeletal muscle volume and mass as the main pathological feature of sarcopenia is associated with osteoporosis. The mechanism is relatively complex, mainly including the influence of the mechanical load of muscle contraction on the mechanical force of bone and the complex and precise endocrine regulation mechanism between muscle and bone [22, 23]. The mechano-regulatory system hypothesis states that muscle contraction directly provides mechanical bone stimulation, promoting osteogenesis [24]. Both muscles and bones have endocrine functions and are regulated by a variety of factors [1].

Several studies have reported the association between sarcopenia and fragility vertebral fracture [25–27]. A survey by Wang et al. showed that sarcopenia was an independent risk predictor of refracture in patients with an osteoporotic vertebral fracture [25]. Tetsuro et al. showed that sarcopenia and decreased calf muscle mass were more common in patients with acute osteoporotic vertebral fractures than in patients without osteoporotic vertebral fractures [27]. Our study found that the PMI of the vertebral fracture group was lower than that of the nonvertebral fracture group in the subjects with nonosteoporosis (*P* value = 0.034). In contrast, the difference in PMI between them in the subjects with osteoporosis was not significant. This study suggests that sarcopenia may be a risk factor for nonosteoporotic vertebral fractures. Therefore, delaying sarcopenia progression and improving skeletal muscle function can reduce the risk of fragility fractures in people without osteoporosis, and these measures may not be beneficial in reducing the risk of fragility fractures in people with osteoporosis.

Our study showed that the height, weight, and BMI of the nonosteoporosis group were higher than those of the osteoporosis group. It suggests that the protective effect of high body weight is probably due to the more significant loading on bones. Other studies suggest that body weight and BMI may be protective factors for BMD [28, 29]. The mechanism may be that decreased sex hormone-binding globulin and increased free sex hormones positively affect BMD in obese patients [30]. In addition, we found that the incidence of osteoporotic and nonosteoporotic fractures was 64% and 52.6%, respectively, and there was no statistical difference (*P* value = 0.117). Several studies have shown that the incidence of fragility fractures with osteopenia and normal bone mass is not low [31–33]. A Korean study showed that more than half of the fragility fractures occurred in women with osteopenia or normal bone mass during the 10-yearfollow-up period [31]. Pasco followed up with 616 postmenopausal women aged 60–94 years. The results showed that 26.9% of fragility fractures were in the osteoporotic group, while 73.1% of fragilities occurred in women without osteoporosis (56.5% in women with osteopenia, 16.6% in women with normal bone mass) [32].

Our study found that combined thoracolumbar fractures were more common in the osteoporosis group (*P* value < 0.001). The progression of osteoporosis is associated with a preferential loss of horizontal trabeculae, the effect of which is reduced ability of the vertebral body to withstand axial compressive forces. The inclination of this bony structure results in vertebral compression fractures, most of which occur in the thoracic and thoracolumbar spine [34].

The study also has some limitations. The study was aimed at middle-aged and elderly women, and the sample was small. The proportion of subjects with osteoporosis, osteopenia, and normal bone mass was not balanced. We combined osteopenia and normal bone mass into the nonosteoporosis group. Therefore, this study cannot more strictly interpret the association of PMI alone with normal bone mass or osteopenia vertebral fracture. This study did not make statistics on the number, location, severity, and time of vertebral fractures. Some subjects had mild lumbar degeneration or spondylolisthesis, which may affect the accuracy of BMD.

## 5. Conclusions

A decrease in height, weight, BMI, bilateral psoas muscle area, and PMI is associated with osteoporosis. Combined thoracolumbar fractures are more common in osteoporosis. Sarcopenia may be an independent risk factor for nonosteoporotic vertebral fractures.

## Figures and Tables

**Table 1:** Baseline data.

	Osteoporosis (*n* = 89)	Nonosteoporosis (*n* = 95)	*t*/chi-square value	*P* value
Age (y)	71.0 ± 6.8	70.0 ± 8.0	0.928	0.355
Age of menopause (y)	49.3 ± 3.3	48.5 ± 4.2	1.330	0.185
Height (m)	1.57 ± 0.05	1.59 ± 0.06	−2.520	0.013
Weight (kg)	57.7 ± 8.5	65.3 ± 9.2	−5.802	<0.001
Body mass index (kg/m^2^)	23.3 ± 3.3	25.7 ± 3.8	−4.663	<0.001
Right psoas muscle area (mm^2^)	446 ± 140	501 ± 142	−2.671	0.008
Left psoas muscle area (mm^2^)	444 ± 114	520 ± 146	−3.915	<0.001
PMI (mm^2^/m^2^)	360.0 ± 98.5	403.0 ± 111.0	−2.773	0.006
L1 BMD (g/cm^2^)	0.726 ± 0.098	0.943 ± 0.140	−12.085	<0.001
L2 BMD (g/cm^2^)	0.745 ± 0.107	1.014 ± 0.142	−14.452	<0.001
L3 BMD (g/cm^2^)	0.803 ± 0.125	1.082 ± 0.151	−13.592	<0.001
L4 BMD (g/cm^2^)	0.836 ± 0.154	1.109 ± 0.141	−12.505	<0.001
Femoral neck BMD (g/cm^2^)	0.638 ± 0.108	0.788 ± 0.104	−9.573	<0.001
Femoral trochanter BMD (g/cm^2^)	0.534 ± 0.097	0.664 ± 0.097	−9.100	<0.001
*T*-score (SD)	−3.4 ± 0.7	−1.3 ± 0.9	−17.213	<0.001
Vertebral fracture	57 (64.0)	50 (52.6)	2.460	0.117
Humerus fracture	1 (1.1)	0	1.073	0.484
Forearm fracture	3 (3.4)	3 (3.2)	0.007	1.000
Femur fracture	3 (3.4)	2 (2.1)	0.278	0.674
Patella fracture	2 (2.2)	0	2.158	0.233

**Table 2:** The relationship between vertebral fracture site and osteoporosis.

	Osteoporosis (*n* = 57)	Nonosteoporosis (*n* = 50)	Chi-square value	*P* value
Thoracic fracture	10 (9.3)	12 (11.2)	0.203	0.653
Lumbar fracture	20 (18.7)	30 (28)	2.61	0.106
Combined thoracolumbar fracture	27 (25.2)	8 (7.5)	12.331	<0.001

## Data Availability

The datasets cannot be made publicly available, and restrictions are applied to the availability of these data. Yihui Zhang should be contacted if someone wants to request the data from this study.

## Abbreviations

PMI: Psoas muscle index
SMI: Skeletal muscle index
CT: Computed tomography
DXA: Dual-energy X-ray absorptiometry
SD: Standard deviation
BMI: Body mass index
BMD: Bone mineral density
T: Thoracic
L: Lumbar
S: Sacral
ROI: Region of interest.
